# The evolving role of cancer cell line-based screens to define the impact of cancer genomes on drug response^?^

**DOI:** 10.1016/j.gde.2013.12.002

**Published:** 2014-02

**Authors:** Mathew J Garnett, Ultan McDermott

**Affiliations:** Cancer Genome Project, Wellcome Trust Sanger Institute Hinxton, Cambridge, United Kingdom

## Abstract

Over the last decade we have witnessed the convergence of two powerful experimental designs toward a common goal of defining the molecular subtypes that underpin the likelihood of a cancer patient responding to treatment in the clinic. The first of these ‘experiments’ has been the systematic sequencing of large numbers of cancer genomes through the International Cancer Genome Consortium and The Cancer Genome Atlas. This endeavour is beginning to yield a complete catalogue of the cancer genes that are critical for tumourigenesis and amongst which we will find tomorrow's biomarkers and drug targets. The second ‘experiment’ has been the use of large-scale biological models such as cancer cell lines to correlate mutations in cancer genes with drug sensitivity, such that one could begin to develop rationale clinical trials to begin to test these hypotheses. It is at this intersection of cancer genome sequencing and biological models that there exists the opportunity to completely transform how we stratify cancer patients in the clinic for treatment.

**Current Opinion in Genetics & Development** 2014, **24**:114–119

This review comes from a themed issue on **Cancer genomics**

Edited by **David J Adams** and **Ultan McDermott**

For a complete overview see the Issue and the Editorial

Available online 5th March 2014

0959-437X/$ – see front matter, © 2014 The Authors. Published by Elsevier Ltd. All rights reserved.

**http://dx.doi.org/10.1016/j.gde.2013.12.002**

## Introduction

All cancers arise due to the acquisition of somatic mutations in their genomes, which fundamentally alter the function of the protein products of key cancer genes [1^•^]. Such mutations are responsible not only for the development of the cancer in the first instance but also for maintaining the proliferation status and evasion of cell death that are the hallmarks of cancer [2]. To date approximately 500 genes have been identified for which mutations (including somatic coding changes and structural rearrangements) have been causally implicated in cancer (http://www.sanger.ac.uk/genetics/CGP/Census/) [3^•^]. Moreover, next-generation sequencing of large numbers of tumours across many tissue types is currently underway as part of the International Cancer Genome Consortium (ICGC) and The Cancer Genome Atlas (TCGA), and we can expect to have within a decade complete catalogues of somatic mutations for many of the most prevalent cancer types (www.icgc.org; http://cancergenome.nih.gov/).

There is an expectation that these studies will reveal genetic dependencies in cancer that can be targeted therapeutically to improve patient survival. Indeed they have begun to reveal pathways and cellular processes that are subverted in cancer and that may be promising drug targets. However, it is also clear that cross-talk between such pathways and compensatory signalling following drug treatment are also present and as such can only be captured by the examination of how cancer cells respond to treatment over time. Such ‘dynamic’ experiments by their nature require biological models, and here we discuss how large-scale cancer cell line models can be used to associate mutated pathways and processes with the likelihood of drug response in cancer patients.

## Cancer genomics and drug response in the clinic

While most of the current treatment regimens for cancer are based on the tissue of origin, the clinical response of cancer patients to treatment with a particular drug is often highly variable. There is a compelling body of evidence, both clinical and experimental, that for an increasing number of drugs used in the clinic the likelihood of a patient's cancer responding to treatment is strongly influenced by alterations in the cancer genome (Table 1) [4–14]. Critically, these genomic changes can be used as molecular biomarkers to identify patients most likely to benefit from a particular treatment. Arguably the most celebrated example of this has been the use of imatinib, a small molecule inhibitor of the ABL1 tyrosine kinase, to target the fusion protein product of the BCR-ABL translocation seen in chronic myeloid leukaemia [15]. More recently, the use of EGFR and ALK inhibitors in lung cancer patients whose tumours harbour EGFR mutations and EML4-ALK rearrangements, respectively, as well as BRAF inhibitors in melanoma has resulted in significantly improved response rates compared to conventional therapies in those subsets of patients [5,6,9]. Equally striking has been the rapid development of targeted therapies compared to the timeline for cytotoxic therapies, best exemplified by the development of ALK inhibitors for lung cancer, which took less than 5 years from initial identification of the EML4-ALK rearrangement as a molecular biomarker to clinical approval of the drug.

**Figure 1:**
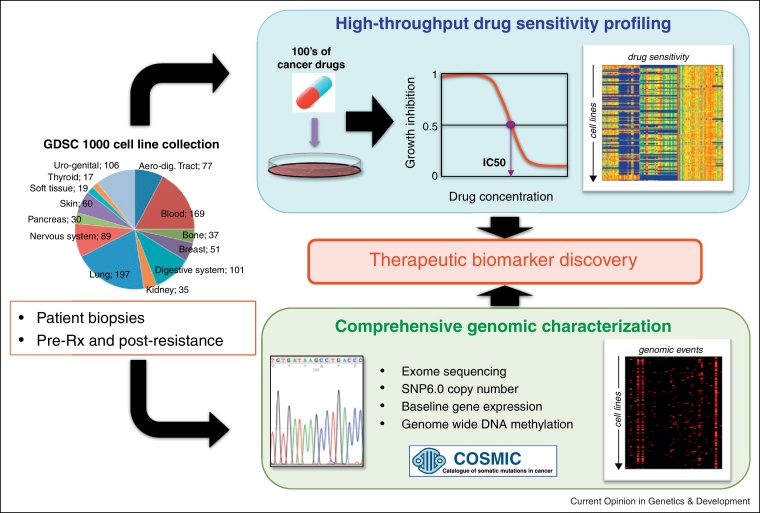
Pharmcogenomic profiling in cancer cells. The genomics of drug sensitivity in cancer project has assembled a panel of >1000 cancer cell lines covering a broad range of tissue types and that have been extensively characterised by exome sequencing, copy number analysis, DNA methylation and mRNA gene expression profiling. Each cell line has drug sensitivity data for a large number of pre-clinical and clinical compounds. These data can be used to identify combinations of mutations, copy number alterations or transcriptional programs that best explain drug response in cancer (www.cancerrxgene.org).

Despite the relative success of these approaches, the number of genomic biomarkers used in the clinic is very small, and the development of new genomic biomarkers has the potential to improve the application of the majority of new and existing therapies. Moreover, even appropriately selected patient populations exhibit a poorly explained range of clinical responses, such as the ~60% response rate in BRAF mutated melanoma patients, which currently limit the effectiveness of even the most targeted approaches. The emergence of clinical resistance appears to be almost a universal feature of targeted therapies, and new clinical strategies incorporating improved biomarkers will be required to monitor, counteract and prevent the emergence of drug resistance. Systematic screens to identify molecular biomarkers to better guide patient therapies, as well as to counter act drug resistance, could have a profound impact on the development of new cancer therapies and ultimately in improving patient outcomes. Therefore, one can begin to imagine how a large panel of cancer cell lines that have been extensively characterised and assayed for their sensitivity to a large collection of pre-clinical and clinical therapeutic agents might enable therapeutic biomarker discovery (Figure 1).

## Cancer cell lines as models for drug biomarker discovery

Immortalised cancer cell lines serve as highly useful and tractable experimental models for cancers in patients and, to a substantial extent, recapitulate in vitro the genetic and biological complexity of cancer. From the establishment of the HeLa cell line almost 50 years ago, they have been the mainstay of biological investigation of human cancer [16]. The current, globally available set of approximately 1000–1500 experimentally usable cancer cell lines constitutes an extraordinarily useful resource that is ubiquitously used in cancer biology and drug development. In particular, cancer cell lines have proven to be invaluable models for cell intrinsic processes and can be used to study the effects on many existing targeted cancer therapies.

Nonetheless, there are specific aspects of cancer biology that are difficult to faithfully model cancer cell lines. These include the effect of tumour–stroma interaction, immune surveillance, invasion and metastasis, angiogenesis and the role of stem cell populations. Moreover, as cell lines can be likened to a snapshot of a tumour, they are not well suited for the study of cancer initiation or progression. This can only be studied properly by employing more complex experimental systems; cell lines have shown themselves to be robust models of cell intrinsic processes. A number of recent publications describing the landscape of driver mutations across a range of cancers have highlighted the mutational heterogeneity present in any given tumour type [17–25]. Similarly, gene expression studies of clinical samples have also defined molecularly defined subgroups within a number of tumour types [26,27,28^•^]. It is entirely plausible that these molecularly defined subgroups will exhibit different biological characteristics including drug response and therefore any screen that utilises cancer cell lines must be of sufficient scale to capture both the tissue-type and genetic diversity of human cancers. Only in this way will it be possible to accurately model the effect of cancer mutations on drug response.

## Large-scale drug screens in cancer cell lines

One of the first systematic efforts to use cancer cell lines to identify biomarkers of drug sensitivity was the NCI-60 panel at the National Cancer Institute in 1990 [29^••^] (http://dtp.nci.nih.gov/branches/btb/ivclsp.html). Although these 60 cell lines have now been screened against many thousands of chemical agents, it has become increasingly clear that much larger numbers of cell lines are required to capture the genetic diversity of human cancer. It is now clear from next-generation sequencing studies that cancers are remarkably heterogeneous and many cancer genes are present in only a fraction of any tumour type. It is therefore likely that hundreds of cancer cell lines would be required to capture this landscape of cancer gene mutations.

To address this need, a Wellcome Trust Sanger Institute and Massachusetts General Hospital collaboration was established in 2009 to screen >1000 cancer cell lines against 400 cancer drugs and to make that data publicly accessible (pharmacologic profiles of 142 cancer drugs screened across 668 cell lines are currently available) (http://www.cancerrxgene.org/) (Figure 1). A similar initiative funded by the pharmaceutical company Novartis at the Broad Institute has profiled 24 cancer drugs across 504 cell lines (http://www.broadinstitute.org/ccle/home). A key element of both endeavours is the detailed genomic, epigenetic and transcriptomic characterisation that has been made possible for these cancer cell lines by advances in next-generation sequencing, such that multi-dimensional signatures of drug response can be derived from such screens and that could be used to stratify patients for clinical trial recruitment or treatment in the clinic. Landmark papers by both these groups recently demonstrated the power of these large screens to identify both novel and previously documented biomarkers of drug response in a completely unbiased fashion [18^••^,30^••^]. It is now feasible to consider profiling all new experimental oncology compounds in such screens in order to develop hypotheses as to mechanisms of activity as well as insights into patient subgroups that may be most likely to respond to treatment in the clinic.

## Tumour organoids as the next generation of cancer cell models

Although the current set of cancer cell lines has demonstrated value when used at sufficient scale to capture the genetic diversity of human cancer, it has a number of drawbacks (Table 2). Foremost among these has been the low success rate in deriving these cell lines from patient biopsies in the past, with the result that some tumour types are very poorly represented (e.g. prostate cancer) and the cell lines available do not completely capture the genetic diversity present in the patient population. It is possible therefore to envisage the ideal scenario for derivation of a new panel of cancer cell lines, where phenotypically stable cells could be generated with high success rates from patient biopsies together with clinical data and where matched normal tissue from the same patient could also be cultured for experimental assays.

Recently the Clevers lab has recently shown that it is possible to establish long-term cultures from a variety of adult mouse and human primary tissues and cancers (‘organoids’), which can be expanded for many months in vitro without genetic or phenotypic changes [31,32^•^]. The essential ingredients of the Matrigel-based 3D organoid cultures are a combination of specific growth factors known to exert strong agonistic effects on critical signalling pathways. Currently, organoid cultures can be made routinely for colon, stomach, and liver [32^•^,33,34]. Protocols for their derivation from pancreas, prostate and lung cancers are also being developed. These organoid cultures will need to be extensively characterised to determine their stability over time and to what degree they match the original cancer biopsy, but the development of this technology raises the possibility of generating a new panel of tumour organoid cultures to replace the current 1000 cancer cell lines that are currently available. These developments are the specific focus of an article in this edition of Current Opinion in Genetics and Development (‘Organoid cultures for the analysis of cancer phenotypes’).

## Concluding remarks

Remarkable advances in DNA sequencing technologies are transforming our ability to define the mutational burden of any given cancer and in the near future these data will become a routine part of the clinical decision-making process to stratify patients for treatment. In order to empower clinicians to interpret how these mutations can affect cancer treatment outcome there will be a continual need for model systems to functionally link these genomic alterations with drug response. Cancer cell lines screened at sufficient scale to capture the existing genetic diversity provide a route into defining the patient subgroups that are more likely to respond to any given therapy. Furthermore, many of the current disadvantages of the current cancer cell lines will potentially be overcome in the near future by their replacement with potentially even larger panels of tumour organoid models. Thus it is likely that such systematic efforts to understand the biology of drug response in cancer will become increasingly important for any new drug in order to better understand the patient subgroup most likely to respond in the clinic. Indeed, one can readily imagine a time in the not too distant future when all new cancer therapeutics will be routinely submitted to such screens and the hypotheses generated used to guide clinical trial design.

## References and recommended reading

Papers of particular interest, published within the period of review, have been highlighted as:

• of special interest
•• of outstanding interest

## Figures and Tables

**Table 1:** FDA-approved targeted cancer drugs in clinical use that are dependent for activity on the presence of a genomic alteration in the patient's tumour. Approved small molecule inhibitors and antibodies targeting specific drug-sensitizing mutations in human cancers. Although these mutations dramatically affect the likelihood of a given patient responding to a particular therapy, it is pertinent to note that in many cases these mutations are only present in a subset of that specific tumour type. Identification of these subgroups using next-generation sequencing technologies will become increasingly important for the management of cancer patients in the clinic

Tumour	Gene (mutation)	Prevalence of gene alteration (%)	FDA-approved drug	Year approved	Therapeutic target	Response rate in mutant tumours (%)	Study
Chronic myeloid leukaemia	BCR-ABL (translocation)	>95	Imatinib	2001	ABL1	>95	Druker *et al.* [4]
Gastrointestinal stromal tumour	KIT (mutation), PDGFRA (mutation)	85 (KIT), 5–8 (PDGFRA)	Imatinib	2002	KIT, PDGFRA	>80	Verweij *et al.* [8]
Non-small cell lung cancer	EGFR (mutation)	10	Gefitinib, erlotinib	2003, 2004	EGFR	70	Mok *et al.* [6]
Chronic myeloid leukaemia (imatinib-resistant)	BCR-ABL (translocation)	>95	Dasatanib	2006	ABL1	>90	Talpaz *et al.* [7]
Breast cancer–node +ve	HER2 amplification	15–20	Trastuzunab	2006	ERBB2	HR 0.48	Perez *et al.* [13]
Melanoma	BRAF (mutation)	40–70	Vemurafenib	2011	BRAF	>50	Chapman *et al.* [20]
Non-small cell lung cancer	EML4-ALK (translocation)	2-7	Crizotinib	2011	ALK	57	Kwak *et al.* [5]
Melanoma	BRAF (mutation)	40–70	Debrafenib	2013	BRAF	52	Hauschild *et al.* [10]
Melanoma	BRAF (mutation)	40–70	Trametinib	2013	MEK1	22	Flaherty *et al.* [11]
Non-small cell lung cancer	EGFR (mutation)	10	Afatinib	2013	EGFR/ERBB2	50	Yang *et al.* [12]
Breast cancer (metastatic)	HER2 amplification	15–20	Trastuzumab	2013	ERBB2	44	Verma *et al.* [14]

*Abbreviations*: KIT, v-kit Hardy-Zuckerman 4 feline sarcoma viral oncogene homologue; PDGFRA, platelet-derived growth factor receptor; alpha polypeptide.

**Table 2:** The advantages and disadvantages of using cancer cell lines to model cancer biology. It is pertinent to note that many of the current disadvantages of established cancer cell lines could be negated through the generation of tumour organoid cancer models using novel tissue culture and sequencing technologies

Advantages	Disadvantages
Cancer is an intrinsic disease of cells.	Some cancer types are very poorly represented as cancer cell lines, for example prostate cancer.
Cancer cell lines are derived from naturally occurring human cancers.	Even for those cancer classes that are represented, there are relatively small numbers available as cancer cell lines.
Cancer cell line resources capture at least some of the cell-of–origin and mutational diversity of cancer.	Cancer cell lines do not reflect the cell-type or tissue architecture of the tissue from which they were derived.
Cancer cell lines are routinely used in drug development.	The available set of cancer cell lines have adapted to culture in multiple different ways. They have been derived over five decades or more in a large number of laboratories under widely differing conditions, and have been grown for widely differing numbers of passages.
Cancer cell lines are tractable for high-throughput analysis as well as gene silencing and overexpression experiments.	The available set of cancer cell lines appears to represent, for many cancer classes, a subset of cases with pre-existing favourable intrinsic features that have allowed establishment in in vitro culture.
	For most cancer cell lines there is little or no clinical or pathological data attached.
	For most cancer cell lines, a normal sample from the same individual is not available and hence we cannot clearly identify the somatic mutations present in the cell line.
	For almost all cancer cell lines, there has not been parallel genomic or other characterisation of the primary cancer from which it was derived in order to assess the degree of similarity (or difference) and the extent to which the line has evolved in vitro.
	The recent explorations of cancer genomes through sequencing, with concomitant discovery of new cancer genes, have revealed how patchy is the recapitulation of key driver events in each cancer type within the current series of cancer cell lines, and how few of the combinations of mutated cancer genes are found therein.

## References

[1•] Stratton M.R., Campbell P.J., Futreal P.A. (2009). The cancer genome. Nature.

[2] Hanahan D., Weinberg R.A. (2011). Hallmarks of cancer: the next generation. Cell.

[3•] Futreal P.A., Coin L., Marshall M., Down T., Hubbard T., Wooster R., Rahman N., Stratton M.R. (2004). A census of human cancer genes. Nat Rev Cancer.

[4] Druker B.J., Talpaz M., Resta D.J., Peng B., Buchdunger E., Ford J.M., Lydon N.B., Kantarjian H., Capdeville R., Ohno-Jones S. (2001). Efficacy and safety of a specific inhibitor of the BCR-ABL tyrosine kinase in chronic myeloid leukemia. N Engl J Med.

[5] Kwak E.L., Bang Y.J., Camidge D.R., Shaw A.T., Solomon B., Maki R.G., Ou S.H., Dezube B.J., Janne P.A., Costa D.B. (2010). Anaplastic lymphoma kinase inhibition in non-small-cell lung cancer. N Engl J Med.

[6] Mok T.S., Wu Y.L., Thongprasert S., Yang C.H., Chu D.T., Saijo N., Sunpaweravong P., Han B., Margono B., Ichinose Y. (2009). Gefitinib or carboplatin-paclitaxel in pulmonary adenocarcinoma. N Engl J Med.

[7] Talpaz M., Shah N.P., Kantarjian H., Donato N., Nicoll J., Paquette R., Cortes J., O’Brien S., Nicaise C., Bleickardt E. (2006). Dasatinib in imatinib-resistant Philadelphia chromosome-positive leukemias. N Engl J Med.

[8] Verweij J., Casali P.G., Zalcberg J., LeCesne A., Reichardt P., Blay J.Y., Issels R., van Oosterom A., Hogendoorn P.C., Van Glabbeke M. (2004). Progression-free survival in gastrointestinal stromal tumours with high-dose imatinib: randomised trial. Lancet.

[9] Chapman P.B., Hauschild A., Robert C., Haanen J.B., Ascierto P., Larkin J., Dummer R., Garbe C., Testori A., Maio M. (2011). Improved survival with vemurafenib in melanoma with BRAF V600 E mutation. N Engl J Med.

[10] Hauschild A., Grob J.J., Demidov L.V., Jouary T., Gutzmer R., Millward M., Rutkowski P., Blank C.U., Miller W.H., Kaempgen E. (2012). Dabrafenib in BRAF-mutated metastatic melanoma: a multicentre, open-label, phase 3 randomised controlled trial. Lancet.

[11] Flaherty K.T., Robert C., Hersey P., Nathan P., Garbe C., Milhem M., Demidov L.V., Hassel J.C., Rutkowski P., Mohr P. (2012). Improved survival with MEK inhibition in BRAF-mutated melanoma. N Engl J Med.

[12] Yang J.C., Hirsh V., Schuler M., Yamamoto N., O’Byrne K.J., Mok T.S., Zazulina V., Shahidi M., Lungershausen J., Massey D. (2013). Symptom control and quality of life in LUX-Lung 3: a phase III study of afatinib or cisplatin/pemetrexed in patients with advanced lung adenocarcinoma with EGFR mutations. J Clin Oncol.

[13] Perez E.A., Romond E.H., Suman V.J., Jeong J.H., Davidson N.E., Geyer C.E., Martino S., Mamounas E.P., Kaufman P.A., Wolmark N. (2011). Four-year follow-up of trastuzumab plus adjuvant chemotherapy for operable human epidermal growth factor receptor 2-positive breast cancer: joint analysis of data from NCCTG N9831 and NSABP B-31. J Clin Oncol.

[14] Verma S., Miles D., Gianni L., Krop I.E., Welslau M., Baselga J., Pegram M., Oh D.Y., Dieras V., Guardino E. (2012). Trastuzumab emtansine for HER2-positive advanced breast cancer. N Engl J Med.

[15] Druker B.J., Guilhot F., O’Brien S.G., Gathmann I., Kantarjian H., Gattermann N., Deininger M.W., Silver R.T., Goldman J.M., Stone R.M. (2006). Five-year follow-up of patients receiving imatinib for chronic myeloid leukemia. N Engl J Med.

[16] Scherer W.F., Syverton J.T., Gey G.O. (1953). Studies on the propagation in vitro of poliomyelitis viruses. IV. Viral multiplication in a stable strain of human malignant epithelial cells (strain HeLa) derived from an epidermoid carcinoma of the cervix. J Exp Med.

[17] Barbieri C.E., Baca S.C., Lawrence M.S., Demichelis F., Blattner M., Theurillat J.P., White T.A., Stojanov P., Van Allen E., Stransky N. (2012). Exome sequencing identifies recurrent SPOP, FOXA1 and MED12 mutations in prostate cancer. Nat Genet.

[18••] Barretina J., Caponigro G., Stransky N., Venkatesan K., Margolin A.A., Kim S., Wilson C.J., Lehar J., Kryukov G.V., Sonkin D. (2012). The cancer cell line encyclopedia enables predictive modelling of anticancer drug sensitivity. Nature.

[19] Berger M.F., Lawrence M.S., Demichelis F., Drier Y., Cibulskis K., Sivachenko A.Y., Sboner A., Esgueva R., Pflueger D., Sougnez C. (2011). The genomic complexity of primary human prostate cancer. Nature.

[20] Chapman M.A., Lawrence M.S., Keats J.J., Cibulskis K., Sougnez C., Schinzel A.C., Harview C.L., Brunet J.P., Ahmann G.J., Adli M. (2011). Initial genome sequencing and analysis of multiple myeloma. Nature.

[21] Ding L., Getz G., Wheeler D.A., Mardis E.R., McLellan M.D., Cibulskis K., Sougnez C., Greulich H., Muzny D.M., Morgan M.B. (2008). Somatic mutations affect key pathways in lung adenocarcinoma. Nature.

[22] Hodis E., Watson I.R., Kryukov G.V., Arold S.T., Imielinski M., Theurillat J.P., Nickerson E., Auclair D., Li L., Place C. (2012). A landscape of driver mutations in melanoma. Cell.

[23] Imielinski M., Berger A.H., Hammerman P.S., Hernandez B., Pugh T.J., Hodis E., Cho J., Suh J., Capelletti M., Sivachenko A. (2012). Mapping the hallmarks of lung adenocarcinoma with massively parallel sequencing. Cell.

[24] Peifer M., Fernandez-Cuesta L., Sos M.L., George J., Seidel D., Kasper L.H., Plenker D., Leenders F., Sun R., Zander T. (2012). Integrative genome analyses identify key somatic driver mutations of small-cell lung cancer. Nat Genet.

[25] Stransky N., Egloff A.M., Tward A.D., Kostic A.D., Cibulskis K., Sivachenko A., Kryukov G.V., Lawrence M.S., Sougnez C., McKenna A. (2011). The mutational landscape of head and neck squamous cell carcinoma. Science.

[26] Curtis C., Shah S.P., Chin S.F., Turashvili G., Rueda O.M., Dunning M.J., Speed D., Lynch A.G., Samarajiwa S., Yuan Y. (2012). The genomic and transcriptomic architecture of 2,000 breast tumours reveals novel subgroups. Nature.

[27] Sadanandam A., Lyssiotis C.A., Homicsko K., Collisson E.A., Gibb W.J., Wullschleger S., Ostos L.C., Lannon W.A., Grotzinger C., Del Rio M. (2013). A colorectal cancer classification system that associates cellular phenotype and responses to therapy. Nat Med.

[28•] Budinska E., Popovici V., Tejpar S., D’Ario G., Lapique N., Sikora K.O., Di Narzo A.F., Yan P., Hodgson J.G., Weinrich S. (2013). Gene expression patterns unveil a new level of molecular heterogeneity in colorectal cancer. J Pathol.

[29••] Shoemaker R.H. (2006). The NCI60 human tumour cell line anticancer drug screen. Nat Rev Cancer.

[30••] Garnett M.J., Edelman E.J., Heidorn S.J., Greenman C.D., Dastur A., Lau K.W., Greninger P., Thompson I.R., Luo X., Soares J. (2012). Systematic identification of genomic markers of drug sensitivity in cancer cells. Nature.

[31] Sato T., Vries R.G., Snippert H.J., van de Wetering M., Barker N., Stange D.E., van Es J.H., Abo A., Kujala P., Peters P.J. (2009). Single Lgr5 stem cells build crypt-villus structures in vitro without a mesenchymal niche. Nature.

[32•] Sato T., Stange D.E., Ferrante M., Vries R.G., Van Es J.H., Van den Brink S., Van Houdt W.J., Pronk A., Van Gorp J., Siersema P.D. (2011). Long-term expansion of epithelial organoids from human colon, adenoma, adenocarcinoma, and Barrett's epithelium. Gastroenterology.

[33] Huch M., Dorrell C., Boj S.F., van Es J.H., Li V.S., van de Wetering M., Sato T., Hamer K., Sasaki N., Finegold M.J. (2013). In vitro expansion of single Lgr5+ liver stem cells induced by Wnt-driven regeneration. Nature.

[34] Barker N., Ridgway R.A., van Es J.H., van de Wetering M., Begthel H., van den Born M., Danenberg E., Clarke A.R., Sansom O.J., Clevers H. (2009). Crypt stem cells as the cells-of-origin of intestinal cancer. Nature.

